# bZIPDB : A database of regulatory information for human bZIP transcription factors

**DOI:** 10.1186/1471-2164-8-136

**Published:** 2007-05-30

**Authors:** Taewoo Ryu, Juhyun Jung, Sunjae Lee, Ho Jung Nam, Sun Woo Hong, Jae Wook Yoo, Dong-ki Lee, Doheon Lee

**Affiliations:** 1 Department of Bio and Brain Engineering, Korea Advanced Institute of Science and Technology, 373-1 Guseong-dong, Yuseong-gu, Daejeon, 305-701, Korea; 2 Department of Chemistry, Pohang University of Science and Technology, Hyo-ja-dong, Nam-gu, Pohang, 790-784, Korea

## Abstract

**Background:**

Basic region-leucine zipper (bZIP) proteins are a class of transcription factors (TFs) that play diverse roles in eukaryotes. Malfunctions in these proteins lead to cancer and various other diseases. For detailed characterization of these TFs, further public resources are required.

**Description:**

We constructed a database, designated bZIPDB, containing information on 49 human bZIP TFs, by means of automated literature collection and manual curation. bZIPDB aims to provide public data required for deciphering the gene regulatory network of the human bZIP family, e.g., evaluation or reference information for the identification of regulatory modules. The resources provided by bZIPDB include (1) protein interaction data including direct binding, phosphorylation and functional associations between bZIP TFs and other cellular proteins, along with other types of interactions, (2) bZIP TF-target gene relationships, (3) the cellular network of bZIP TFs in particular cell lines, and (4) gene information and ontology. In the current version of the database, 721 protein interactions and 560 TF-target gene relationships are recorded. bZIPDB is annually updated for the newly discovered information.

**Conclusion:**

bZIPDB is a repository of detailed regulatory information for human bZIP TFs that is collected and processed from the literature, designed to facilitate analysis of this protein family. bZIPDB is available for public use at .

## Background

Transcription factors (TFs) are responsible for gene expression in every living organism. The bZIP family shares a basic region and a leucine zipper domain. Homo/hetero-dimerization between family members is possible through the leucine zipper domain, and the proteins bind target promoters via the basic amino acid-rich region [1]. The bZIP TFs play essential roles in several processes in eukaryotic cells, from early development to tumorigenesis. For example, JUN is an oncogene that affects diverse cellular processes including proliferation, differentiation and apoptosis [2], while CEBPA is a well-known regulator of hepatocyte and adipocyte development [3].

With the assistance of high-throughput technology, such as microarray technology, several researchers have attempted to decipher the regulatory networks of bZIP TFs [4-7]. However, this type of evaluation is largely dependent on manual literature search, which is time-consuming and incomplete. While a number of the binding proteins or target genes of bZIP TFs can be retrieved from HPRD or TRANSFAC [8,9], the currently available data are relatively limited, and do not necessarily cover the entire cellular network. For gene transcription, multiple steps are required, i.e., signaling cascade of multiple proteins, interactions between TFs and other proteins (such as RNA polymerase) or other TFs, and TF binding to DNA in the proper orientation. Thus, to elucidate the entire regulatory network, extensive data on the above processes must be amassed and processed.

To facilitate our understanding of these proteins, we have generated a bZIPDB database containing regulatory network information on the human bZIP TF family. In particular, we focus on the signaling protein-TF interactions, TF-TF interactions, and TF-target gene interactions that are important for regulatory network analysis with high-throughput technology.

## Construction and content

The aim of bZIPDB is to accumulate known regulatory information on human bZIP TFs, particularly protein-protein and protein-DNA interactions. A list of human bZIP TFs with the appropriate synonyms is documented on our website. For database construction, public literature dealing with human bZIP TFs, including official symbols and synonyms, was initially obtained from PubMed [10] using web queries. The PubMed IDs of 2,498 papers for 49 TFs were stored and arranged in our internal web-based curation system via an automated process. Regulatory information was processed and saved under a suitable format in the database by experts.

**Figure 1:**
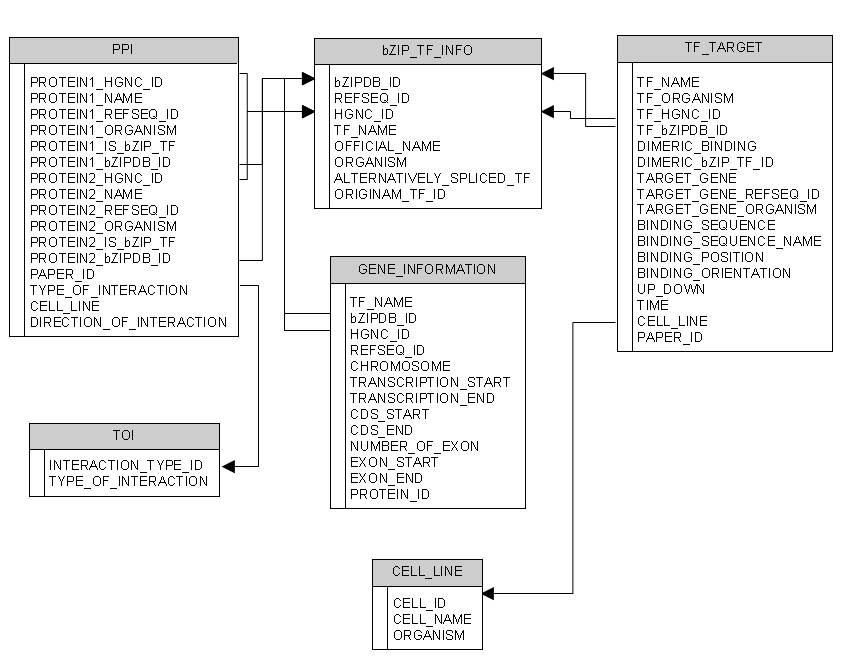
**Schematic diagram of bZIPDB**. Underlying relational database schema for bZIPDB.

The regulatory network of the bZIP family is grouped into six tables, depending on specific attributes. The system architecture of bZIPDB is depicted in Figure 1, and details of the attributes are recorded on our website.

• **bZIP_TF_INFO: **Basic information on human bZIP TFs, such as bZIPDB ID, official symbol, RefSeq ID and transcript variants.

• **GENE_INFORMATION: **Information of the chromosomal loci and exons of human bZIP TFs.

• **PPI: **Protein-protein interactions between bZIP TFs and other proteins.

• **TF_TARGET: **bZIP TF-target gene promoter interactions.

• **CELL_LINE: **Experimental cell lines and their origin.

• **TOI: **Types of protein-protein interactions.

For 49 human bZIP TFs, bZIPDB ID was assigned on the basis of the distinct mRNA transcript. Since alternatively spliced or transcribed products encoded by the same gene have different biochemical properties [11], we assigned different IDs to each bZIP TF and its transcript variant, as reflected in PPI and TF_TARGET tables.

In the construction of a protein-protein interaction table, information on interaction types, directions of interactions, and cell lines is collected in addition to the identities of interacting proteins. While several databases have focused on the direct binding of proteins acting as complexes [8,12], cellular protein networks also consist of other interaction types, such as phosphorylation and SUMOylation. Functional association, which means that both proteins are present in the same pathway, is another important interaction type in transcriptome analysis, which basically assumes that coregulated genes share similar roles [13,14]. These interaction types are specified in the TOI table. 'Direction of interaction' indicates that one protein affects the activity of another protein, i.e. upstream or downstream in the signaling pathway. RefSeq ID for each protein is appended as a crosslink to NCBI. The organism from which the protein originates is also added as an attribute, since researchers often use proteins from different sources. Experimental cell lines are additionally classified as an important attribute, since they originate from different organisms and tissues and therefore have a distinct genomic context, which affects protein-protein interactions (described in the CELL_LINE table).

The target genes of TF are less well characterized, compared to protein-protein interactions. For transcription, TFs bind to specific DNA sequences in the proper orientation, which is influenced by nearby proteins, such as histone or other TFs. While known TF-target gene relationships from a few databases have been used as positive examples, their number is too limited to constitute a positive dataset. For example, 51 mammalian target genes of human JUN protein are recorded in TRANSFAC 10.4 [9], while we have identified 88 in bZIPDB. Hence, several findings, including TFs, targets, results of transcription (i.e. activation or repression), binding sequences, binding positions, and cell lines, have been incorporated in the database. Moreover, as bZIP TFs often act via homo/hetero-dimerization between family members, the dimerizing partner is included if specified in the literature. The database statistics are summarized in Table 1.

Another unique aspect of bZIPDB is the compilation of regulatory information for particular cell lines. Each cell line originates from different organisms or tissues, which maintain unique genetic and epigenetic compositions, hence affecting various cellular interactions. Therefore, careful consideration is required when data from several resources are used in conjunction. To clarify the distinctive cellular networks and accuracy of interactions, bZIPDB provides the list of regulatory interactions conducted in a particular cell type. Table 2 summarizes the popularly used cell lines and number of interactions listed in bZIPDB.

In addition to protein-protein and protein-DNA interaction data, genomic information, such as chromosomal locus and exon/introns, synonyms and functional annotation, was obtained from Entrez [15] and the Gene ontology consortium [16].

## Utility and Discussion

**Figure 2:**
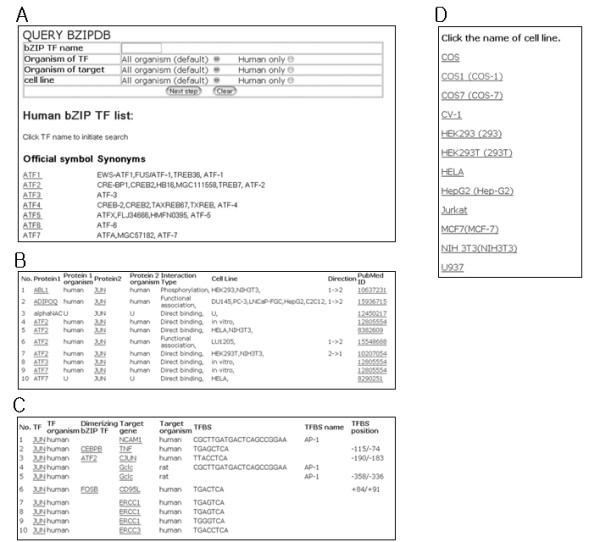
**Queries and results of bZIPDB**. (A) The 'Search bZIPDB' menu shows the query box and human bZIP TF list. Typing in or clicking on the names of bZIPs allows search initiation. (B) Protein-protein interactions of JUN. Interaction partners and types, interaction directions and other data are shown. (C) Protein-DNA interactions of JUN. JUN regulates target genes by binding to the TF binding site (TFBS). (D) 'Cellular Network' page. By clicking on the cell name, scientists can access interaction data for the specified cell line.

bZIPDB provides a convenient search engine with which users can explore the database either by typing bZIP TF names within the query box or by clicking on the listed names (Figure 2A). The known human bZIP TFs are listed on the 'search bZIPDB' page, according to the alphabetical order of the official symbol. The 'official symbol' is the approved gene name by public databases, such as NCBI and HGNC [17]. Synonyms collected from NCBI and HGNC are recorded next to the official symbol. On the input form, users can type in the individual bZIP TF name (either the official symbol or synonyms). By default, the results pages return all records in bZIPDB, regardless of the organism of TF, target gene or cell line. However, users can restrict the organism category to humans. For convenience, bZIPDB allows searches by simply clicking on the bZIP TF name with default options.

The results page returns basic information, such as names, RefSeq ID, chromosomal locus and exon/intron positions of the bZIP TF protein examined. By clicking on the 'Protein interaction' or 'Target genes' menu on the right side of the results page, researchers can recover detailed reports on protein-protein or protein-DNA interactions of bZIP TF, respectively (Fig. 2B and 2C) to facilitate further analysis. These include official symbol, organism, interaction type, TF binding sites and positions, cell lines, and PubMed id, among other information. An external link to NCBI RefSeq and PubMed is provided for each interaction and gene. If the organism is not specified in the literature, it is impossible to ascertain gene identity (RefSeq ID). In this case, the positions are denoted 'U' (unspecified). A bZIPDB report of human JUN is shown as an example (Figs. 2B and 2C). In bZIPDB, 148 protein-protein and 88 protein-DNA interactions are accessible, while 110 protein-protein and 51 protein-DNA interactions are retrieved from HPRD and TRANSFAC, respectively. Moreover, these two databases do not use official symbols in the search and result pages, and are therefore difficult to exploit in terms of bZIP TF analysis. The official symbols are very important, since they greatly facilitate integration between various information sources, e.g., microarray and interaction data. bZIPDB contains more information on human bZIP TFs than other databases, and is therefore more useful for the analysis of these proteins.

Interactions within specific cell lines can be viewed on the 'Cellular Network' page (Figure 2D). In total, 12 popular cell lines are listed. By clicking on the name of the cell line, researchers may retrieve associated interactions from the database. The result format is similar to query results of individual bZIP TF proteins. Data in bZIPDB are available in a tab-delimited format on the 'Download' page. Interaction data subsets (protein-protein and protein-DNA) are also available in either the tab-delimited or the simple interaction format (SIF), supported by Cytoscape [18], a visualization and integration tool.

bZIPDB aims to serve as a portal for researchers studying the human bZIP TF family. To date, the database has focused on amassing the relevant literature data. However, the updated version of bZIPDB will provide other types of data. One data category involves the potential target genes of bZIP TFs, which are computationally predicted using phylogenetic footprinting and motif search algorithms [19,20]. Another is genome-wide mRNA expression profiles, which are accumulated in public databases, such as NCBI GEO [21]. Differential expression patterns of bZIP TFs will be collected along with relevant information, such as experimental conditions and cell lines. Since integration of interaction data from different databases is an important issue, collected data will be subjected to the HUPO PSI's molecular interaction format [22]. Finally, the database will be updated annually.

## Conclusion

bZIPDB contains extensive information on human bZIP TFs, such as manually curated protein-protein and protein-DNA interactions, genomic information, synonyms, and gene ontology. Moreover, this novel database provides classified interaction data for popularly used cell lines, leading to a clearer picture of the cell type-specific subnetwork. Thus, bZIPDB constitutes a valuable resource to facilitate comprehensive understanding and analysis of the cellular network of human bZIP TFs.

## Availability and requirements

bZIPDB home page : 

Operating systems(s): Linux

Programming language: Java

License: the database is freely available to academic and non-academic users.

## List of abbreviations

Basic region-leucine zipper (bZIP), transcription factors (TF), transcription factor binding site (TFBS), protein-protein interaction (PPI)

## Authors' contributions

TR designed the database and drafted the manuscript. JJ, SL, HJN, SWH and JWY participated in data curation. Dong-ki Lee and Doheon Lee supervised the work.

## Figures and Tables

**Table 1:** The statistics of bZIPDB

Category	Number of records
TFs	49
Protein-protein interactions	721
Direct binding	516
Phosphorylation	47
Functional association	143
Other interactions	15
TF-target gene relations	560
Activation	279
Repression	57

A list of interaction types with the number of records

**Table 2:** Cell lines and summary of interactions in bZIPDB

cell line	organism	# of protein-protein interaction	# of TF-target interaction
COS1	monkey	10	14
COS7	monkey	23	9
HEK293	human	38	19
HEK293T	human	42	17
HELA	human	48	46
HepG2	human	23	89
Jurkat	human	19	22
MCF7	human	5	12
NIH3T3	mouse	19	14
U937	human	14	7

Ten popularly used cell lines with the originating organisms and number of interactions (listed in bZIPDB).
